# External Validation and Recalibration of a Mortality Prediction Model for Patients with Ischaemic Stroke

**DOI:** 10.3390/jcm12227168

**Published:** 2023-11-18

**Authors:** Juan Manuel García-Torrecillas, María Carmen Lea-Pereira, Laura Amaya-Pascasio, Carmen Rosa-Garrido, Miguel Quesada-López, Fernando Reche-Lorite, Mar Iglesias-Espinosa, Adrián Aparicio-Mota, José Galván-Espinosa, Patricia Martínez-Sánchez, Miguel Rodríguez-Barranco

**Affiliations:** 1 Emergency and Research Unit, Torrecárdenas University Hospital, 04009 Almería, Spain; 2 Centro de Investigación Biomédica en Red de Epidemiología y Salud Pública (CIBERESP), 28029 Madrid, Spain; miguel.rodriguez.barranco.easp@juntadeandalucia.es; 3 Instituto de Investigación Biosanitaria ibs.GRANADA, 18012 Granada, Spain; 4 Servicio de Medicina Interna, Hospital Universitario de Poniente, 04700 Almería, Spain; mariac.lea.sspa@juntadeandalucia.es; 5 Stroke Centre, Department of Neurology, Torrecárdenas University Hospital, 04009 Almería, Spain; laura.amaya.pascasio@gmail.com (L.A.-P.); miguel.quesadalopez@gmail.com (M.Q.-L.); patrinda@ual.es (P.M.-S.); 6 FIBAO, Hospital Universitario de Jaén, Servicio Andaluz de Salud, 23007 Jaén, Spain; crosa@fibao.es; 7 Departamento de Matemáticas, Universidad de Almería, 04120 Almería, Spain; freche@ual.es; 8 Unidad de Investigación Biomédica, Hospital Universitario Torrecárdenas, 04009 Almería, Spain; aaparicio@fibao.es; 9 FIBAO, Hospital Universitario Torrecárdenas, Servicio Andaluz de Salud, 04009 Almería, Spain; jgalvan@fibao.es; 10 Faculty of Health Sciences, Health Research Center (CEINSA), University of Almeria, Carretera de Sacramento s/n, 04120 Almeria, Spain; 11 Escuela Andaluza de Salud Pública (EASP), 18011 Granada, Spain

**Keywords:** ischaemic stroke, predictive model, validation study, epidemiology

## Abstract

Background: Stroke is a highly prevalent disease that can provoke severe disability. We evaluate a predictive model based on the Minimum Basic Data Set (MBDS) compiled by the Spain Health Ministry, obtained for the period 2008–2012 for patients with ischaemic stroke in Spain, to establish the model’s validity and to optimise its calibration. The MBDS is the main clinical-administrative database for hospitalisations recorded in Spain, and to our knowledge, no predictive models for stroke mortality have previously been developed using this resource. The main study aim is to perform an external validation and recalibration of the coefficients of this predictive model with respect to a chronologically later cohort. Material and Methods: External validation (testing the model on a different cohort to assess its performance) and recalibration (validation with optimisation of model coefficients) were performed using the MBDS for patients admitted for ischaemic stroke in the period 2016–2018. A cohort study was designed, in which a recalibrated model was obtained by applying the variables of the original model without their coefficients. The variables from the original model were then applied to the subsequent cohort, together with the coefficients from the initial model. The areas under the curve (AUC) of the recalibration and the external validation procedure were compared. Results: The recalibrated model produced an AUC of 0.743 and was composed of the following variables: age (odds ratio, OR:1.073), female sex (OR:1.143), ischaemic heart disease (OR:1.192), hypertension (OR:0.719), atrial fibrillation (OR:1.414), hyperlipidaemia (OR:0.652), heart failure (OR:2.133) and posterior circulation stroke (OR: 0.755). External validation produced an AUC of 0.726. Conclusions: The recalibrated clinical model thus obtained presented moderate-high discriminant ability and was generalisable to predict death for patients with ischaemic stroke. Rigorous external validation slightly decreased the AUC but confirmed the validity of the baseline model for the chronologically later cohort.

## 1. Introduction

Cerebrovascular disease, in general, and ischaemic stroke, in particular, present a major burden of disease worldwide. Stroke is not only one of the main causes of global mortality; it also provokes the loss of many years of healthy life due to disability. Overall, stroke is the second cause of mortality and the leading cause of disability; in industrialised countries, it is the third cause of mortality [1,2].

More than 60% of patients with stroke present an ischaemic profile, and many are not suitable for reperfusion treatment due to the time elapsed since the onset of symptoms an inadequate response to pre-hospital management of the stroke, or insufficient healthcare resources (which vary enormously among hospitals, regions, and countries).

In general, recent systematic reviews have found that the incidence of stroke is decreasing, thanks to improvements in the control of cardiovascular risk factors such as diabetes, smoking, and hypertension. However, rising life expectancies and rates of survival could lead to an increase in global prevalence, especially among the elderly [2,3,4].

The design and application of a predictive model of mortality risk is a valuable means of enhancing the quality of healthcare for patients with cerebral infarction, as such a model would enable clinical teams to stratify the severity and prognosis of the condition and then adapt clinical pathways and action protocols accordingly. Several models have been proposed for evaluating the risk of mortality and sequelae [5,6,7,8]. Some have been applied to clinical-administrative registers and databases [9,10,11,12,13], but their utility is limited because these records do not contain the specific variables found in the main scale used worldwide for assessing the risk and severity of stroke, i.e., the National Institutes of Health Stroke Scale (NIHSS) [14].

Over the last decade, there has been a significant increase in the development and publication of predictive models in virtually all medical fields. Nevertheless, significant gaps remain with respect to external validation reports. Only 5% of published models are accompanied by some form of validation, and insisting on this procedure could help bridge the gap between model development and implementation. In addition, model designs are sometimes inadequate, and important considerations such as discriminant capacity, model calibration, and how to deal with missing data are omitted [15,16].

One of the major problems encountered in this respect is the lack of external validation of published models. A predictive model is an equation that estimates the individual risk of presenting a certain outcome based on certain predictors or variables of the individual. These models are very useful for personalised medicine, making it possible to adopt individualised therapeutic measures and facilitate risk stratification [17,18].

However, a model may provide excellent predictions when applied to the individuals from whom it was developed but poorly when applied to an external cohort [19]. Therefore, the performance of the model must be tested on a new set of patients to confirm its satisfactory performance; this process is termed external validation [17]. It can be applied to a cohort that is chronologically earlier or later than the context of the original model cohort (“temporal validation”) or by any alternative procedure that ensures that the validation cohort differs substantially from the development cohort [18,20,21,22].

Lea-Pereira et al. (2022) [11] developed a mortality estimation model for use at first hospital admission based on the Minimum Basic Data Set (MBDS) (compiled by the Spanish Health Ministry) of patients admitted in Spain for non-reperfused stroke during the period 2008–2012. This model had moderate-high discriminant capacity (AUC: 0.742, 95% CI [0.74–0.75]), good visual calibration according to the representation of the risk deciles, and included most of the sociodemographic and clinical variables that are usually recorded during hospital admission in Spain.

This instrument, which we term the Baseline Model (BM), is a useful auxiliary resource for patients who are not eligible for reperfusion treatment. It enables the risk to be stratified and helps emergency healthcare staff and neurologists adopt the most appropriate response.

The MBDS is a clinical-administrative database that is of compulsory application in the Spanish National Health System. It contains administrative, sociodemographic, and comorbid information on patients and details of the procedures performed during admission. To our knowledge, no predictive model of post-stroke mortality has previously been developed in Spain [23,24].

The current study has two main objectives. Firstly, to characterise the BM and maximise its performance by applying it to a chronologically later population (the validation cohort, VC), corresponding to the period 2016–2018. This cohort was constructed using the ICD-10 classification rather than the ICD-9MC used in the BM. This new approach allows us to obtain recalibrated coefficients for an optimised model (termed the recalibrated model, RM). Secondly, we evaluate the performance of the BM by applying its original variables and coefficients to the VC, thus determining whether it remains valid for the second population (this process is termed external validation).

## 2. Materials and Methods

### 2.1. Design

In this analytical observational study, we perform an external validation and subsequent recalibration of the coefficients of the original variables from a prior model [11] used to predict the mortality at first admission of patients with non-reperfused ischaemic stroke.

External validity was assessed by applying the original WB—the original variables and coefficients—to the subsequent cohort. The subsequent recalibration process consisted of maximising the coefficients of each variable to achieve the best possible performance of the model in the new sample.

### 2.2. Information Source

The BM was constructed from all episodes of hospitalisation for ischaemic stroke when the patient was ineligible for reperfusion (diagnosis-related group, DRG, 14) in Spain during the period 2008–2012. The information was obtained from the Spanish Ministry of Health, Consumer Affairs and Social Welfare and was coded using the 9th edition of the International Classification of Diseases, Clinical Modification (ICD-9MC). The model was obtained using binary logistic regression, and the variables included were age, sex, 30-day readmission status, chronic ischaemic heart disease, diabetes mellitus, hypertension, dyslipidaemia, heart failure, and symptoms suggestive of posterior circulation stroke. More detailed information on the BM can be found in the original publication [11].

To obtain the external validation and recalibration of model coefficients, the BM was applied to a validation cohort. The VC consisted of all episodes of non-reperfused ischaemic stroke (DRG 45) for which patients were hospitalised in Spain during the period 2016–2018. This VC was also obtained from the MBDS, and the information was coded using ICD-10.

### 2.3. Variables

For both prediction models, the rate of in-hospital mortality at first admission was taken as the dependent variable. In other words, the models focus on patients who died during admission for ischaemic stroke and did not receive reperfusion treatment.

The remaining variables were taken as predictor or independent variables and were used to optimise and develop the models. The sociodemographic variables included were age, sex, and location of hospital admission, while the relevant comorbidities considered included ischaemic heart disease, chronic obstructive pulmonary disease, atrial fibrillation, hypertension, diabetes, and hyperlipidaemia (Table 1). Also evaluated were the length of stay at first admission, the number of diagnoses at discharge (NDD) as a proxy variable for diagnostic effort and comorbidities, and the number of procedures performed prior to discharge (NPD) as a proxy for treatment effort.

As in the development of the BM [11], the VC database was subjected to a moderate degree of data purging to exclude outliers for the variable “length of hospital stay”. For this purpose, we used the formula T2 = Q3 + 1.5 (IQR) where Q is the third quartile, IQR is the interquartile range, and T2 is the maximum length of stay above which a data point is considered an outlier. The latter value was taken as 21 days.

### 2.4. Method and Statistical Analysis

After a descriptive, exploratory consideration of the main study variables, a bivariate analysis was performed to detect associations between mortality and each of the independent variables. These associations are expressed as the unadjusted Odds Ratio (ORu) together with the corresponding 95% confidence interval and level of statistical significance.

The external validation and model recalibration procedures were then carried out. For external validation, the BM variables and their original regression coefficients were applied to the VC. The performance of each model was assessed according to its AUC and 95% CI.

In the second stage of our analysis, the coefficients of the variables included in the BM were recalibrated to optimise the model. The BM variables were then applied to the VC using a binary logistic regression procedure in which the dependent variable was mortality. This process generated new coefficients that formed the basis for the recalibrated model (RM). The discriminant capacity of this model was determined by calculating the area under the ROC curve, and it was calibrated by graphically representing the risk deciles obtained by the Hosmer–Lemeshow test. Finally, the RM was evaluated by various machine learning procedures (Random Forest, Tree, Neural Network, and Gradient Boosting). In each case, the model’s AUC, accuracy (i.e., percentage of cases in which the model was correct), F1 (combination of the precision and completeness metrics), precision (i.e., positive predictive value), and recall (equivalent to the standard concept of sensitivity) were determined.

The analysis concluded with the internal validation of the RM by a cross-validation procedure to reveal the existence or otherwise of overfitting and “excessive optimism” in the new model. For this purpose, the external cohort was divided into two subsets: training and test). A repeated cross-validation method was then used with k = 5 and 10 repetitions in the training subset. The model was then trained on the test subset. The corresponding AUC was obtained in each case.

## 3. Results

### 3.1. Descriptive Study

The analysis was based on 147,092 hospitalisation episodes that took place during the period 2016–2018 (of these patients, 53.5% were men, n = 78,712). The patients had a mean age of 74.85 years, the mean hospital stay was 6.99 days, and the in-hospital mortality rate was 10.6%. The baseline characteristics of the sample and the main comorbidities recorded are shown in Table 1.

With respect to the patients who survived hospital admission, the average age of those who died before discharge was 9.5 years lower. Moreover, this population recorded less procedural effort (NPD 2.00 vs. 2.80) and a shorter average length of stay (6.26 vs. 7.08) (Table 2).

The bivariate analysis based on the ORu showed that the main variables associated with in-hospital mortality were female sex (OR 1.774, 95% CI 1.715–1.835), ICU admission (OR 3.070, 95% CI 2.900–3.250), COPD (OR 1.173, 95% CI 1.102–1.248), chronic respiratory failure (OR 4.741, 95% CI 3.838–5.857)), atrial fibrillation (OR 2.293, 95% CI 2.216–2.372), chronic kidney disease (OR 1.835, 95% CI 1.735–1.922) and ischaemic heart disease (OR 1.243, 95% CI 1.181–1.308). Hypertension (OR 0.966, 95% CI 0.932–1.001), dyslipidaemia (OR 0.506, 95% CI 0.467–0.549), and symptoms of posterior circulation stroke (SPCS) (OR 0.647, 95% CI 0.602–0.695) were protective factors. The existence of a previous transient ischaemic attack was not associated with mortality (Table 2).

### 3.2. External Validation

**Figure 1:**
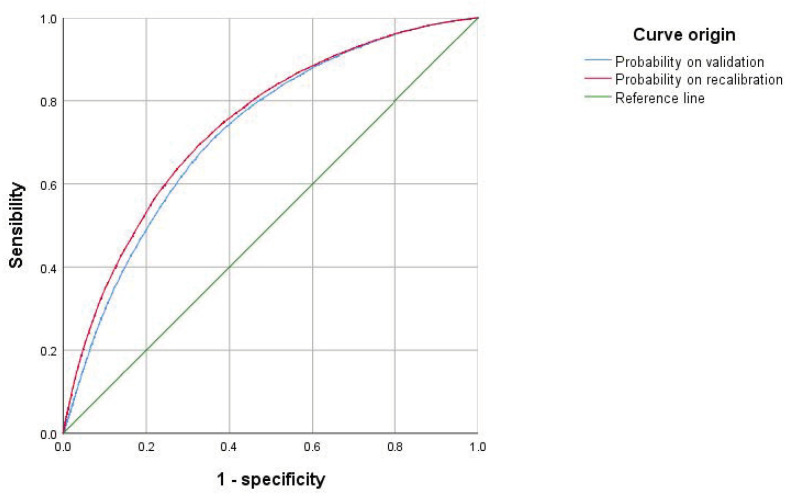
ROC curves corresponding to the external validation of the model. AUC on “Probability on validation”: 0.726; AUC on “Probability on recalibration”: 0.743.

Application of the full BM (original variables and coefficients) to the VC produced an AUC of 0.726, 95% CI: 0.722–0.730. Recalibration showed an AUC of 0.743, 95% CI: 0.739–0.747. Figure 1 shows the differences observed in the amplitude of the CIs between the validation and the recalibrated models, together with the ROC curve for each case.

### 3.3. Recalibrated Model

Application of the BM variables (Table 3) to the external cohort generated a recalibrated model (RM) with updated coefficients (Table 4). The readmission variable was not considered because this term did not exist in the ICD-10 coding of the external cohort, and diabetes was depreciated due to loss of significance in the model.

The logistic regression performed to obtain the RM showed that atrial fibrillation (OR 1.41), heart failure (OR 2.13), and ischaemic heart disease (OR 1.19) were the main predictor variables (Table 4 and the “Probability on recalibration” curve in Figure 1). This model had a discriminant capacity, estimated using the AUC, of 0.743, 95% CI: 0.739–0.747. The application of data science metrics revealed high scores for accuracy and AUC-ROC and low ones for recall and F1-score. The precision (0.725) could only be determined by gradient boosting (Table 5).

**Figure 2:**
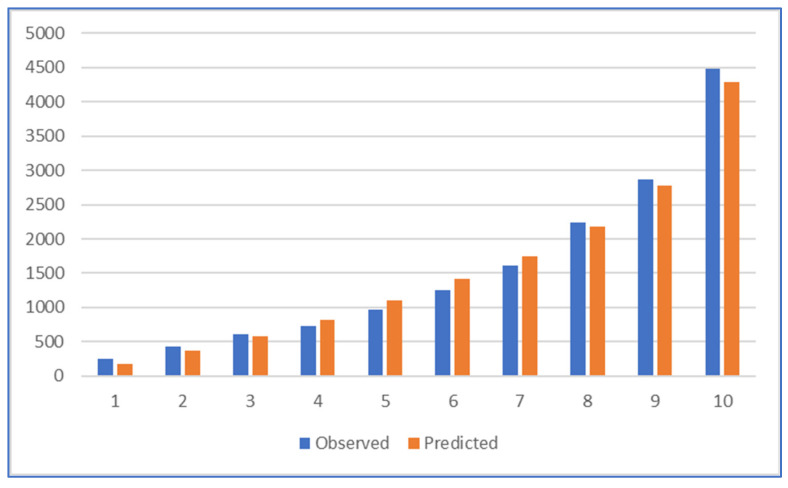
Calibration graph of the Recalibrated Model according to risk deciles.

The results of the Hosmer–Lemeshow test of calibration were significant, but the visual representation of the observed cases versus those expected in the risk deciles was more strongly discernible (Figure 2).

### 3.4. Internal Validation of the Recalibrated Model

The RM contained all of the BM variables except readmission (which does not exist in ICD-10MC) and diabetes mellitus (due to lack of statistical significance).

For validation, the external cohort was divided into two subsets (training test). The repeated cross-validation method was then used, with k = 5 and 10 repetitions first for the training subset and then for the test subset. The following AUC values were obtained: training subset 0.743, 95% CI: 0.738–0.747; test subset 0.743, 95% CI: 0.734–0.752.

### 3.5. Importance of the Predictors in the RM

**Figure 3:**
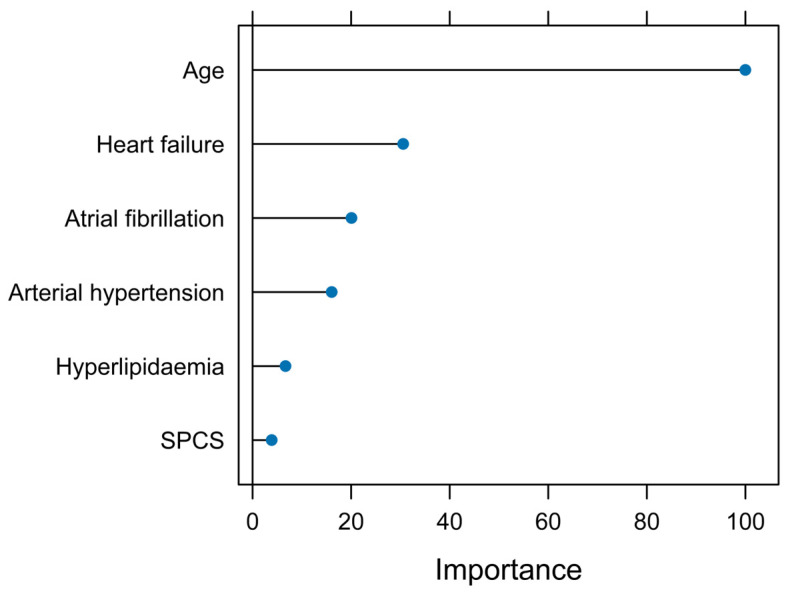
Relative importance of the variables in the recalibrated model.

According to the coefficients obtained by the K-fold validation method, the most important predictors were age and heart failure (Figure 3).

## 4. Discussion

### 4.1. Findings

The aim of this study was to perform the external validation of a predictive model for mortality from ischaemic stroke using a chronologically later cohort (2016–2018), termed the validation cohort (VC). Subsequently, the coefficients were recalibrated to generate an optimised model with better performance and predictive capacity, termed the recalibrated model (RM). The external validation produced the following results: when the baseline model (BM) was applied to the VC, the model’s discriminant capacity was reduced. Thus, the AUC fell from 0.742 to 0.726. This decrease in performance is common when a prognostic model is applied to a large group of cases that have not participated in its development [25]. Nevertheless, the performance of the predictive model remained in the moderate-high range of possible values. In relation to all of the above, we can draw the clinical conclusion that the predictive model of ischaemic mortality thus obtained is generalisable to a chronologically later, unrelated population.

The next stage of our analysis was to obtain an MR equipped with new coefficients and with a discriminant capacity similar to that of the original model. The AUC for the RM was 0.743, versus 0.742 for the original BM and 0.726 for the VC. These results show that the RM model outperformed the BM in mortality estimation when applied to a cohort coded according to the ICD-10 classification. Moreover, the RM maintained its discriminant power despite the severe difficulties provoked by the change in the form of coding. The internal validation of the RM via a cross-validation procedure corroborated its robustness and revealed an acceptable absence of overfitting [26].

The inclusion of age, sex, atrial fibrillation, heart failure, and ischaemic heart disease as predictor variables in the RM is consistent with the literature, as these factors have previously been identified as important in assessing the mortality risk of patients with ischaemic stroke [27].

In short, the study achieved our two main objectives, generating a model that was validated both externally and internally, with its coefficients recalibrated and with moderate-high discriminant capacity.

### 4.2. Comparison with Previous Studies

The observed decrease in discriminant capacity during external validation was only 0.016 points (from the original AUC of 0.742 to 0.726 in the VC). This result was to be expected for several reasons. Firstly, the model’s application to a cohort of cases totally unrelated to those for which it was originally developed will normally provoke a decrease in discriminant capacity. Other relevant factors in this decrease include the differences between the two versions of the ICD used (ICD-9MC and ICD-10) and, most especially, the elimination of the “readmission” variable from the latter, as this variable is highly sensitive to clinical severity in this set of patients. Overall, the results obtained in the external validation were consistent with those found in previous studies, and the discriminant capacity obtained was moderate-high.

Some of the predictor variables used in the present study have been considered not only in studies of mortality prediction but also in those aiming to predict the risk of ischaemic stroke. One such study considered a cohort of 4503 patients whose cases were followed up for two years, during which 22 new episodes of ischaemic stroke were reported. This study modelled the risk of ischaemic stroke using (among others) the variables age, sex, exercise, food, BMI, and visceral adiposity index. An AUC of 0.79 was calculated for the risk of ischaemic stroke [28]. This study, despite the important difference in the variable to be predicted, highlighted the need to consider common variables such as age and sex, both of which are highly significant to the predictive capacity of each model.

In a recent study, Huang et al. (2023) [13] evaluated the in-hospital mortality of elderly patients treated in the ICU for ischaemic stroke and developed predictive models of 28-day mortality using naive Bayes methodology, logistic regression, and XG Boosting. The best results were obtained with XG Boosting (AUC 0.733), although this value was slightly below that obtained by us, according to classical logistic regression (0.743). The mortality rate was 13.3% versus the 10.6% obtained in our study. These results are reasonably similar, taking into account that our case mix was not exclusive to the ICU. When the results were evaluated by logistic regression, as in the central element of our research, the AUC of Huang’s model dropped to 0.627, much lower than was obtained by our model. However, both models presented high specificity and accuracy.

In any case, the evidence strongly suggests that the model we propose is very suitable for evaluating the severity and prognosis of patients hospitalised for stroke using the National Institute of Health Stroke Scale (NIHSS), which is rarely available in the Spanish Primary Care Clinical Database (PCCDB) but provides very reliable results even in retrospective studies [29].

Other researchers have focused on predicting the severity and risk of mortality during admission for ischaemic stroke, in line with our own study goals. In one notable case [30], the US National Inpatient Sample (NIS) database was analysed using the main clinical-administrative variables recorded in ICD-10 and for the same time period (2016–2018). This paper concluded that in-hospital mortality due to ischaemic stroke was significantly associated with atrial fibrillation, carotid stenosis, diabetes, heart failure, and ischaemic heart disease. In this study population, mortality was higher in women than in men and also when ischaemic heart disease was associated with stroke. Both of these aspects are consistent with our results. The latter findings are not particularly novel. During the period 2001–2007, studies using the database for the “Get With the Guidelines-Stroke” programme [8] obtained the first validated models for predicting in-hospital mortality. Their results, despite the time that has since elapsed, were similar to later findings, especially in terms of the comorbidities associated with mortality, and are totally consistent with those of the current project, although the discriminant capacity was substantially lower (AUC 0.72 in the validation sample, which increased to 0.85 when the NIHSS scale was included). When not only mortality but also clinical deterioration at admission was examined (using nomograms obtained by logistic regression), the results were completely concordant [31].

Various other studies are also consistent with the findings we present. Some are particularly interesting as they are based on analogous data sources (i.e., clinical-administrative records). Thus, Joundi et al. [32] developed a predictive model using only data from clinical-administrative records and validated a 30-day mortality model using a specific indicator of stroke severity (termed the “passive surveillance stroke severity indicator”). The inclusion of this indicator improved the discriminant capacity of the models from 0.72 to 0.80. When the study data were linked to a clinical database, it was found that the inclusion of the NIHSS scale further optimised the model one year after the stroke, although the study findings were qualified by the fact that this variable was only available in cases where some type of reperfusion had been performed. In addition, Waddell et al. [33] and Aylin et al. [24] have both shown that the use of data from electronic records and large databases could provide a good approximation in the initial assessment of stroke severity. Using the latter sources, each of these studies proposed a 30-day mortality model. In both cases, the AUC was similar to our own finding (0.76).

Another interesting approach is that of the “PLAN score” developed by O’Donnell et al. [12], using a model based on the variables present at the time of hospital admission. The model is composed of nine comorbidities, of which data for five (age, preadmission dependence, cancer, heart failure, and atrial fibrillation) were known prior to admission, while the other four (level of consciousness, neurological deficit, aphasia, and neglect) were determined at the time of admission. This model presented good discriminant capacity for 30-day mortality and predicted the Rankin score at discharge acceptably well.

### 4.3. Study Limitations

#### 4.3.1. Revised ICD Classification

A fundamental aspect of the limitations and difficulties generated by the VC for the period 2016–2018 is that of the change in the International Classification of Diseases from ICD-9MC (used in preparing the BM) to ICD-10. As a result, the model was validated not only on a chronologically later cohort but also using a much larger and more complex coding system, ICD-10. The revised classification system incorporates new concepts, and many chapters have been comprehensively changed or are completely new. The purpose of this more detailed coding, of course, is to better describe the medical conditions encountered and the clinical procedures used. Among other benefits, ICD-10 significantly improves the coding of the topography of the lesion and enables new diagnostic and therapeutic procedures to be incorporated. To sum up, in the present study, the model was validated using a database in which, while the basic principles were maintained, substantial modifications had been made to the coding methodology, thus presenting a significant challenge to the validation process [34].

#### 4.3.2. PCCDB per se and the NIHSS Scale

In general, using the Spanish PCCDB or similar large database presents a major problem because most of the cases included do not present clinical variables of interest. As concerns the NIHSS scale in particular, a very important limiting element is the absence of some variables that are necessary for our purposes. Quite clearly, the score recorded on this scale is strongly associated with the patient’s status at discharge and with the severity of the underlying condition [32,35,36]. Accordingly, the non-availability of this information represents an important problem. A large administrative database, such as the NIS [9] or the one consulted in the present study, provides important benefits due to the large sample size, the valuable content of sociodemographic variables and comorbidities, and the possibility of focusing the study on a chronologically extensive period. However, like the CMBD, these sources lack certain clinical variables, specifically those that would facilitate the construction of the NIHHS scale. This circumstance makes it difficult to adjust for case severity. Another handicap is the non-inclusion of the modified Rankin score at 90 days [9,37]. The NIHSS score, evaluated on large US databases [35], was under-reported, as has been observed in earlier population-based studies (the score was only obtained for 1 of every 7 cases of stroke). Nevertheless, this score is of undoubted value for estimating the severity of stroke and the patient’s risk of mortality [35].

Sung et al. [38] derived a scale (the Stroke Severity Index, SSI) exclusively from administrative claims-based data for patients with ischaemic stroke, but the results obtained were not especially striking, with the incorporation of the SSI producing only a slight increase in discriminant capacity, compared to previous models. An interesting alternative was proposed by Simpson et al., who developed a severity score based on administrative data available at the moment of hospital discharge [39]. This score was used to predict the NIHSS via a model that provided high discriminant capacity (AUC 0.83), which could be useful for post-discharge risk adjustment models based on administrative data. In any case, estimates of intrinsic NIHSS values obtained from PCCDBs are still extremely uncommon; accordingly, the NIHSS continues to be a valuable means of assessing patient health status.

On the other hand, PCCDBs also have limitations in terms of completeness; they tend to be much more heterogeneous than databases and registries designed to collect data on specific pathologies and frequently present under-recording bias. Such information sources are usually fairly accurate regarding administrative data but suffer from significant heterogeneity in terms of quality with respect to the coding of discharge circumstances and comorbidities, among other questions [40,41]. Finally, PCCDBs may provide a useful complement to clinical databases. Moreover, their use could promote ongoing quality control, a point in their favour with respect to purely administrative information sources [24].

#### 4.3.3. Database Imbalance

When the target variable represents a small minority of the overall population (as in the present case, a very low prevalence of death), this imposes certain limitations that must be considered in any evaluation of the model. The main problem arising from this is that the model is biased towards predicting the majority class (living patients) rather than the minority one (the patients who die). Despite this difficulty, the model constructed, in addition to providing high discriminant capacity and good visual calibration, is particularly useful for assessing low-risk patients when the negative predictive value is very high. Although this may be a limitation, it is also advantageous in the initial assessment of low-risk patients. This is a well-known problem when the analyst has to consider a very large database with a class imbalance in the variable to be predicted. This issue could be usefully examined in future research using subsampling techniques in the majority class.

### 4.4. Strengths of the Study

In the proposed model, a large database and a three-year study period are considered. This increases its statistical power and enhances the external validity for predicting mortality risk for ischaemic stroke patients for whom reperfusion is not indicated. The model provides a valuable tool for emergency and neurology personnel to make informed decisions, particularly for low-risk patients. Furthermore, it can be used in conjunction with the NIHSS scale, contributing to the development of precision medicine.

Finally, we believe that this type of model and the decisions derived from its application contribute to a more efficient use of resources and help optimise hospital attention for stroke patients.

## Figures and Tables

**Table 1:** Descriptive variables (n = 147,092).

Quantitative, mean ± sd	
Age (years)	74.85 ± 13.34
NDD	8.40 ± 3.80
NPD	2.72 ± 0.53
**Qualitative, n(%)**	
Female sex	68,380 (46.49)
Mortality	15,638 (10.6)
COPD	10,091 (6.9)
Ischaemic heart disease	15,296 (10.4)
Arterial hypertension	102,028 (69.4)
Obesity	11,365 (7.7)
Renal insufficiency	15,452 (10.5)
Atrial fibrillation	40,047 (27.2)
Diabetes	43,857 (29.8)
Heart failure	7673 (5.2)
Basilar arterial stenosis	11,724 (8.0)

NDD: Number of diagnoses at discharge; NPD: Number of procedures prior to discharge. COPD = Chronic obstructive pulmonary disease.

**Table 2:** Bivariate study. Factors associated with hospital mortality.

Qualitative Variables							
		Total		Exitus			
		n (%)		n (%)	ORu	95% CI OR	*p*
Sex	Men	78,712 (53.5)		6382 (8.1)	1		
	Women	68,380 (46.5)		9256 (13.5)	1.774	1.715; 1.835	<0.001
Year	2016	47,637 (32.4)		5258 (11)	1		
	2017	48,912 (33.3)		5073 (10.4)	0.993	0.895; 0.972	0.010
	2018	50,548 (34.4)		5307 (10.5)	0.945	0.908; 0.984	0.060
ICU	No	135,261 (92.00)		13,280 (9.8)	1		
	Yes	6982 (4.70)		1749 (25.1)	3.07	2.900; 3.250	<0.001
Hypertension	No	45,069 (30.6)		4894 (10.9)	1		
	Yes	102,028 (69.4)		10,744 (10.5)	0.966	0.932; 1.001	0.060
Dyslipidaemia	No	136,025 (92.5)		14,985 (11)	1		
	Yes	11,072 (7.5)		653 (5.9)	0.506	0.467; 0.549	<0.001
COPD	No	146,721 (99.7)		15,503 (10.6)	1		
	Yes	376 (0.3)		135 (35.9)	1.173	1.102; 1.248	<0.001
Chronic respiratory failure	No	107,050 (72.8)		8809 (8.2)	1		
	Yes	40,047 (27.2)		6829 (17.1)	4.741	3.838; 5.857	<0.001
Atrial fibrillation	No	100,997 (68.37)		10,926 (10.8)	1		
	Yes	43,857 (29.8)		4518 (10.3)	2.293	2.216; 2.372	<0.001
Diabetes	No	133,259 (90.6)		14,130 (10.6)	1		
	Yes	13,838 (9.4)		1508 (10.9)	0.947	0.913; 0.982	0.003
Prior TIA	No	131,645 (89.5)		13,043 (9.9)	1		
	Yes	15,452 (10.5)		2,595 (16.8)	1.031	0.975; 1.091	0.285
Chronic kidney disease	No	135,373 (92)		14,777 (10.9)	1		
	Yes	11,724 (8)		861 (7.3)	1.835	1.753; 1.922	<0.001
SPCS	No	131,801 (89.6)		13,709 (10.4)	1		
	Yes	15,296 (10.4)		1,929 (12.6)	0.647	0.602; 0.695	<0.001
Ischaemic heart disease	No	146,721 (99.7)		15,503 (10.6)	1		
	Yes	376 (0.3)		135 (35.9)	1.243	1.181; 1.308	<0.001
**Quantitative Variables**							
		**N**	**Mean**	**SD**	**Diff of Means**	**95% CI Interval**	***p***
Age	Survive	15,638	73.84	13.331			
	Death	131,459	83.360	9.913	−9.542	−9.696; −9.353	<0.001
Length of stay	Survive	15,638	7.080	4.504			
	Death	13,1459	6.260	4.797	0.822	0.747; 0.897	<0.001
NDD	Survive	15,638	8.270	3.743			
	Death	131,459	9.531	4.114	−1.261	−1.324; −1.198	0.124
NPD	Survive	15,638	2.800	2.531			
	Death	131,459	2.001	2.333	0.798	0.759; −0.837	<0.001

ICU: Intensive care unit; COPD, Chronic obstructive pulmonary disease; TIA: Transient ischaemic attack; SPCS: symptoms of posterior circulation stroke. NDD: Number of diagnoses at discharge; NPD: Number of procedures prior to discharge.

**Table 3:** Logistic equation corresponding to the initial (baseline) model.

Exitus	OR	95% CI		SD	*p*
		Lower	Upper		
Age	1.069	1.067	1.072	0.001	<0.001
Female sex	1.202	1.149	1.257	0.023	<0.001
Readmission (Yes)	2.008	1.862	2.165	0.038	<0.001
Ischaemic heart disease (Yes)	1.342	1.227	1.467	0.046	<0.001
Hypertension (Yes)	0.726	0.695	0.759	0.023	<0.001
Diabetes (Yes)	1.105	1.054	1.158	0.024	<0.001
Atrial fibrillation (Yes)	1.537	1.471	1.607	0.023	<0.001
Dyslipidaemia (Yes)	0.638	0.606	0.671	0.026	<0.001
Heart failure (Yes)	1.518	1.421	1.622	0.034	<0.001
SPCS (Yes)	2.639	2.071	3.364	0.124	<0.001

AUC: 0.742, 95% CI 0.737–0.747; Pearson’s X2 test: 0.176; SPCS: Symptoms of posterior circulation stroke.

**Table 4:** Logistic equation corresponding to the recalibrated baseline model.

Exitus	OR	95% CI		SD	*p*
		Lower	Upper		
Age	1.073	1.070	1.075	0.001	<0.001
Female sex	1.143	1.102	1.185	0.019	<0.001
Ischaemic heart disease (Yes)	1.192	1.129	1.257	0.027	<0.001
Hypertension (Yes)	0.719	0.692	0.747	0.019	<0.001
Atrial fibrillation (Yes)	1.414	1.363	1.466	0.018	<0.001
Dyslipidaemia (Yes)	0.652	0.600	0.709	0.042	<0.001
Heart failure (Yes)	2.133	2.016	2.258	0.029	<0.001
SPCS (Yes)	0.755	0.701	0.813	0.038	<0.001

AUC: 0.743, 95% CI 0.739–0.747. SPCS: Symptoms of posterior circulation stroke.

**Table 5:** Recalibrated model and Data Science metrics.

Model	AUC	Accuracy	F1-Score	Precision	Recall
Logistic Regression	0.743	0.893	0.011	0.381	0.006
Tree	0.739	0.894	0.022	0.641	0.011
Random Forest	0.761	0.894	0.039	0.592	0.020
Neural Network	0.747	0.894	0.004	0.492	0.002
Gradient Boosting	0.747	0.894	0.004	0.725	0.002

## Data Availability

The data presented in this study are available on request from the corresponding author. The data are not publicly available due to ethical restrictions.
